# Supplementary material for: Humanized Ovarian Cancer Patient-Derived Xenografts for Improved Preclinical Evaluation of Immunotherapies
Source: Cancers (Basel). 2022 Jun 23;14(13):3092. doi: 10.3390/cancers14133092 (PMC9265069; doi:10.3390/cancers14133092)
Supplement: Supplementary file 1 [file cancers-14-03092-s001.zip › cancers-1746340 supplementary material.pdf]

Table S1. 34-marker panel for mass cytometry: Metal-conjugated antibodies were purchased from Fluidigm, BioLegend and Abcam. Five monoclonal antibodies (*) were conjugated in-house to the metal isotopes using the X8 polymer as described by the manufacturer (Fluidigm).

Tag	Metal	Anti-human mAb	Clone	Catalog #	Provider	Dilution
89	Y	CD45	HI30	3089003B	Fluidigm	1:200
141	Pr	CD326	9C4	3141006C	Fluidigm	1:3200
142*	Nd	CD303	201A	354215	BioLegend	1:50
143	Nd	CD117	104-D2	3143001B	Fluidigm	1:800
145	Nd	CD4	RPA/T4	3145001C	Fluidigm	1:200
146	Nd	CD8	RPA/T8	3146001C	Fluidigm	1:200
147	Sm	CD20	2H7	3147001C	Fluidigm	1:600
148	Nd	CD16	3G8	3148004C	Fluidigm	1:200
149	Sm	CD25	2A3	3149010C	Fluidigm	1:200
151	Eu	CD123	6H6	3151001B	Fluidigm	1:200
152	Sm	CD95/Fas	DX2	3152017C	Fluidigm	1:200
153	Eu	CD7	6B7	3153014B	Fluidigm	1:400
154	Sm	CD163	GHI/61	3154007C	Fluidigm	1:100
155	Gd	PD-1	EH12.2H7	3155009B	Fluidigm	1:200
156	Gd	CD86	IT2.2	3156008C	Fluidigm	1:100
158*	Gd	CD335	BAB281	331902	BioLegend	1:200
159[+]	Tb	PD-L1	29E.2A3	3159029B	Fluidigm	1:200
159[#]	Tb	CCR7	Go43H7	3159003C	Fluidigm	1:75
160	Gd	CD14	M5E2	3160001C	Fluidigm	1:100
161	Dy	CTLA-4	14D3	3161004B	Fluidigm	1:200
162	Dy	CD11c	Bu15	3162005C	Fluidigm	1:600
163	Dy	CD56	NCAM16.2	3163007C	Fluidigm	1:800
164	Dy	CD45RO	UCHL1	3164007C	Fluidigm	1:200
165	Ho	CD127	A019D5	3165008C	Fluidigm	1:100
166	Er	CD34	581	3166012B	Fluidigm	1:800
167	Er	CD27	O323	3167002C	Fluidigm	1:100
169	Tm	CD33	WM53	3169010B	Fluidigm	1:200
170	Er	CD3	UCHT1	3170001B	Fluidigm	1:1600
171*	Yb	CD62L	DREG-56	304835	BioLegend	1:200
172*	Yb	CD73	259D/C7	130451	Abcam	1:400
173	Yb	CD141	1A4	3173002C	Fluidigm	1:400
174	Yb	HLA-DR	L243	3174001C	Fluidigm	1:800
175	Lu	mCD45**	30-F11	3175010C	Fluidigm	1:400
176*	Yb	CD1c	L161	331502	BioLegend	1:1600
209	Bi	CD47	CC2C6	3209004B	Fluidigm	1:200

*in-house conjugated, **anti-mouse monoclonal antibody, [+]only in tumor samples, [#] only in blood samples.

Suppl. Figure S1

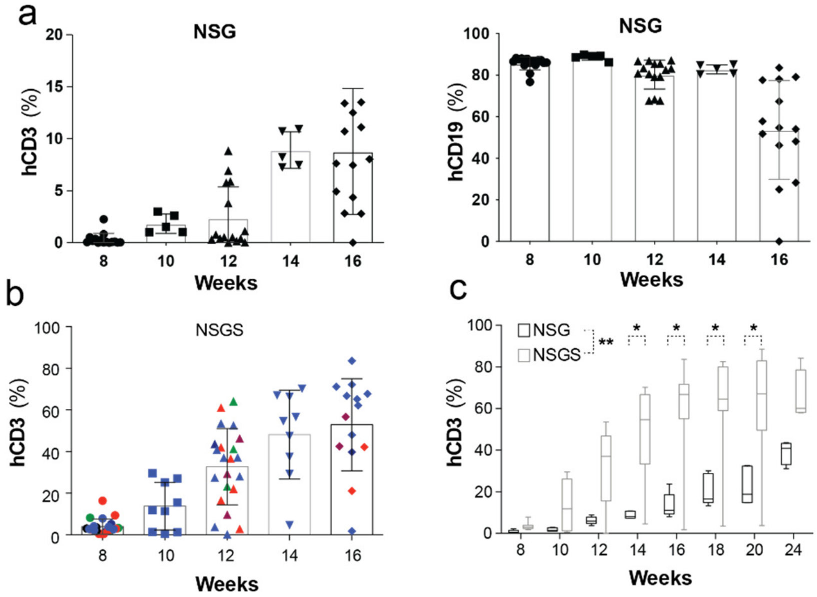

Figure S1. T cell reconstitution in NSG and NSGS mice over time. **(a)** Representative composite data of T cell (left) and B cell (right plot) development representative in NSG mice over time. **(b)** T cell development in hu NSGS mice of five different cord blood donors over time, plotted as mean with SD; each color representing one donor. **(c)** Blood samples of NSG (n = 5) and NSGS (n = 9) mice were drawn biweekly. The frequency of $CD45^{+}CD3^{+}$ hematopoietic cells in the mouse blood was analyzed longitudinally. Multiple t-tests were between NSG and NSGS mice were performed for each time point using the Holm-Sidak method (*$p \leq 0.001$). A Wilcoxon matched pairs signed rank test was performed to assess the difference in T cell reconstitution between NSG and NSGS (**$p = 0.0078$). P values < 0.05 were regarded as statistically significant.

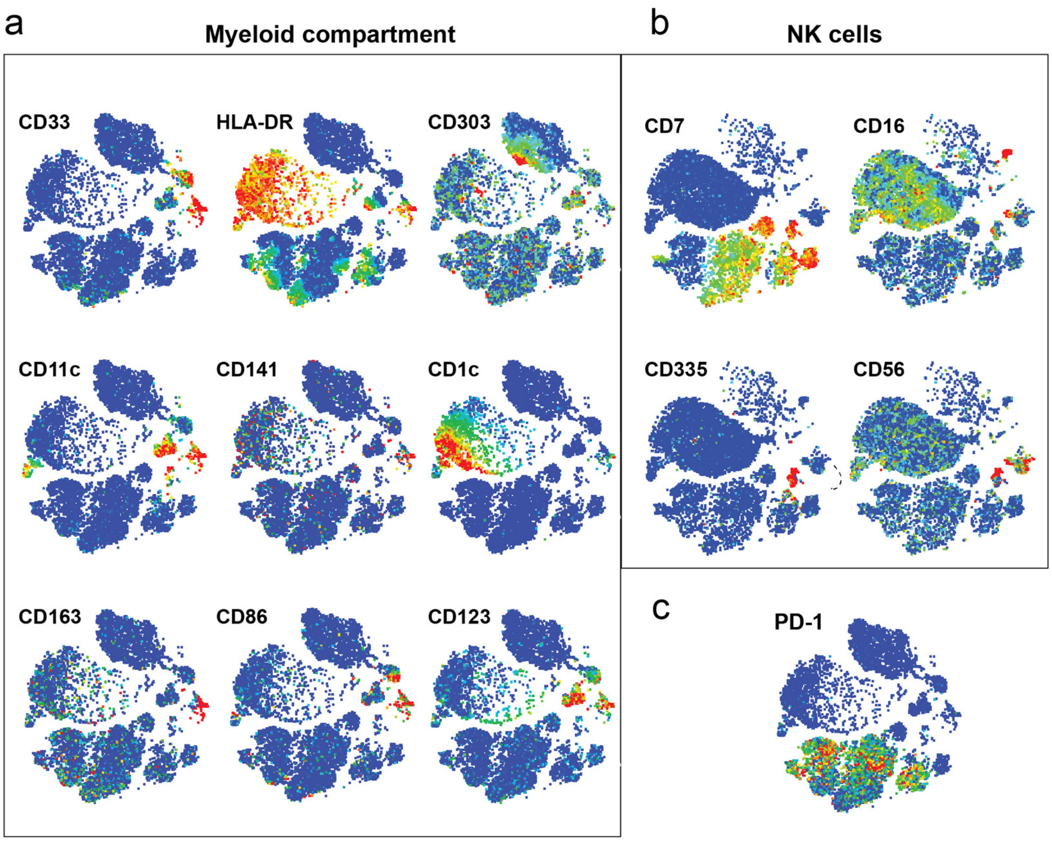

Figure S2. Characterization of immune cells in the blood of NSG and NSGS mice. (a) Reconstituted immune systems in NSG and NSGS mice were analyzed by mass cytometry. viSNE dot plots of NSGS mouse blood colored by myeloid lineage and differentiation marker intensity. (b) viSNE dot plots of NSG mouse blood colored by NK cell surface marker intensity. (c) Expression of PD-1 shown in a viSNE map of NSGS mouse blood samples. All marker expression levels are represented from high (red) to low (blue).

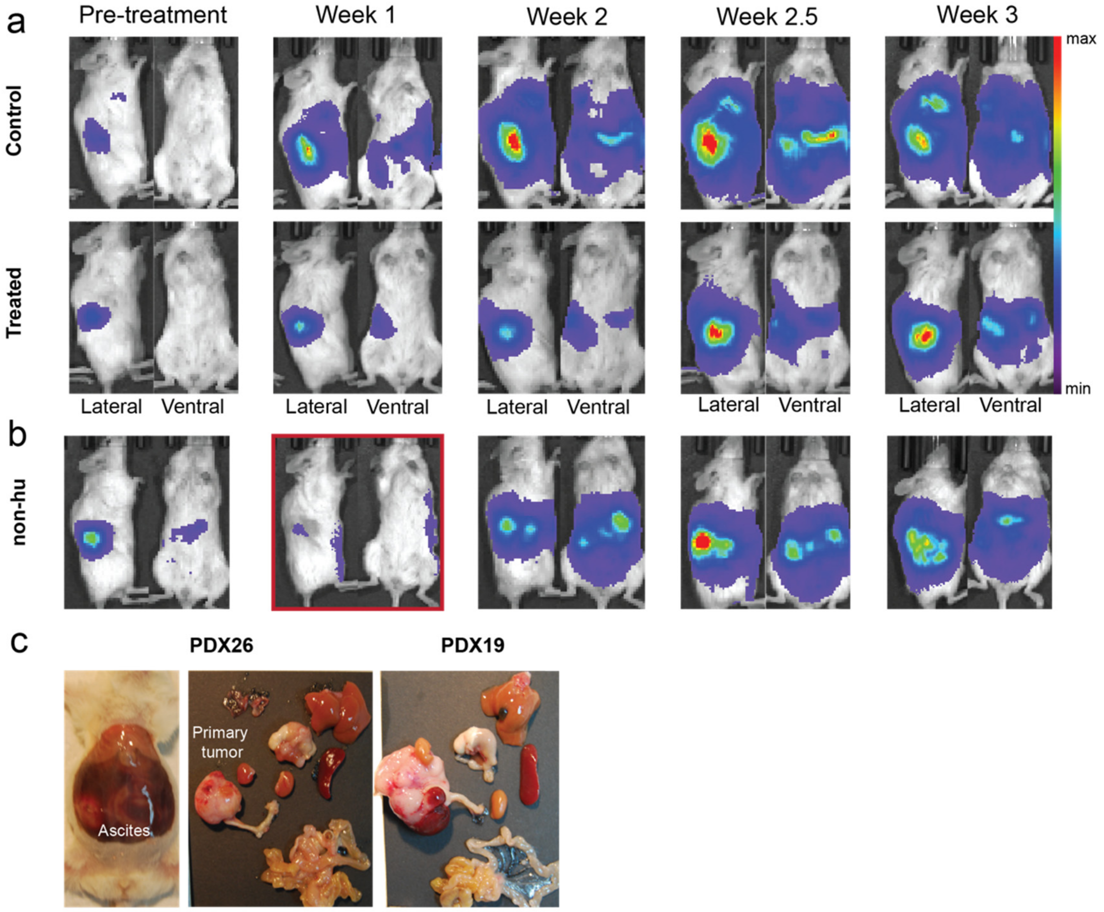

Figure S3. Tumor growth in hu OV-90 xenograft and hu PDX models. **(a)** Disease progression was followed by bioluminescence imaging before and during treatment with nivolumab in hu OV-90^{luc+} xenograft models **(b)** and compared to non-hu OV-90^{luc+} xenograft models treated with nivolumab. Bioluminescence signal is expressed as average radiance (p/s/cm^2/sr) and all images are visualized with the same scale from minimum to maximum. Red-framed images indicate acquisition errors. (c) Disease dissemination in hu PDX26 and PDX19 mice.

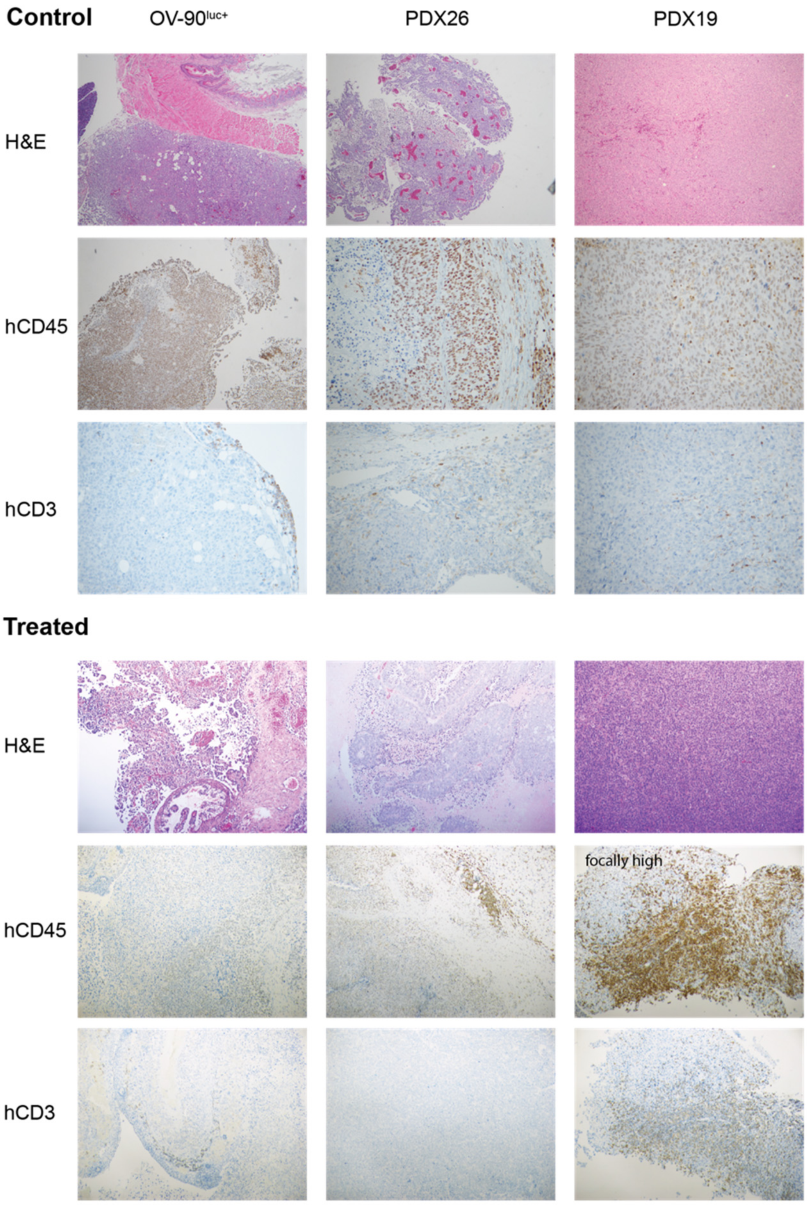

Figure S4. Evaluation of tumor immune cell infiltration by immunohistochemistry. Tumor tissue sections of treated and untreated hu OV-90^{luc+}, hu PDX26 and hu PDX19 models were stained with hematoxylin and eosin (H&E), human CD45 and human CD3.

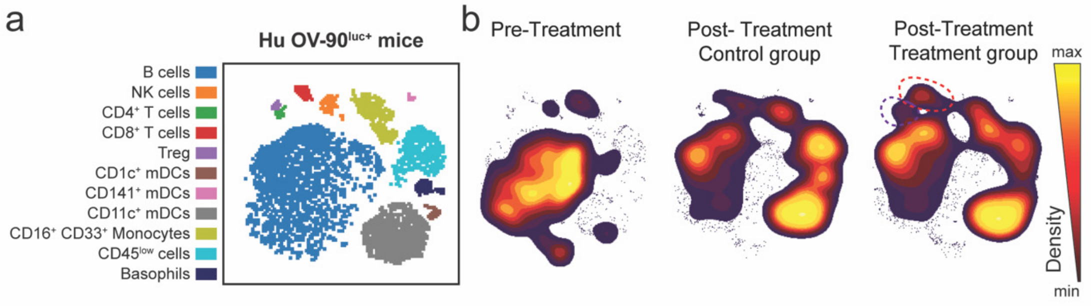

Figure S5. Peripheral immune cell characterization after nivolumab treatment. **(a)** Overlay of manually gated cell clusters on a viSNE map from OV-90^{luc+} xenograft humanized mouse blood. **(b)** Contour density plots of viSNE maps showing the frequency of human immune cell populations in the blood of humanized OV-90^{luc+} mice before and after treatment. $CD8^{+}$ T cells are marked with a dashed red circle, $CD4^{+}$ T cells are marked with a dashed purple circle. Density is represented by a purple (low-density cell population) to yellow (high-density cell population) gradient.
